# The prevalence of comorbidities in COPD patients, and their impact on health status and COPD symptoms in primary care patients: a protocol for an UNLOCK study from the IPCRG

**DOI:** 10.1038/npjpcrm.2016.69

**Published:** 2016-11-17

**Authors:** Björn Ställberg, Pedro Teixeira, Coert Blom, Karin Lisspers, Ioanna Tsiligianni, Rachel Jordan, Miguel Román, Nanne Kleefstra, Oleksii Korzh, David Price, Niels Chavannes

**Affiliations:** 1 Department of Public Health and Caring Sciences, Family Medicine and Preventive Medicine, Uppsala University, Uppsala, Sweden; 2 ICVS/3B's—PT Government Associate Laboratory, Life and Health Sciences Research Institute (ICVS), School of Health Sciences, University of Minho, Braga, Portugal; 3 Stichting Zorgdraad Foundation, Oosterbeek, The Netherlands; 4 Asites Rural Practice, Heraklion, Greece; 5 Institute of Applied Health Research, University of Birmingham, Birmingham, UK; 6 Mallorca Research Institute IdisPa, IBSalut, Palma, Spain; 7 Department of Internal Medicine, University of Groningen and University Medical Center Groningen, Groningen, The Netherlands; 8 Department of Family Medecine, Kharkov Medical Academy, Kharkov, Ukraine; 9 Academic Primary Care, Division of Applied Health Sciences, University of Aberdeen, Aberdeen, UK; 10 Department of Public Health and Primary Care, Leiden University Medical Center, Leiden, The Netherlands

## Background

Chronic obstructive pulmonary disease (COPD) is an important cause of morbidity and mortality with high social and economic costs. The prevalence of COPD has been reported to vary between 6 and 26.1% worldwide.^1^ COPD has also been associated with a high prevalence of one or more comorbid conditions, which have an impact on health status and mortality.^2–5^ Although several diseases have been studied as COPD comorbidities^6,7^ few studies have looked at the issue of multimorbidity in COPD.^8–10^

COPD, like other chronic disorders, has been associated with comorbidities that increase in number and severity with age, and are more prevalent among deprived social groups.^5,8^ There is evidence that comorbidities increase the risk for exacerbations, reduce health status, and increase the risk of mortality.^5,8^ COPD guidelines (e.g., GOLD recommendations) still consider the diagnosis and management of comorbidities from an individual disease point of view.^11^ Consequently, health services focus on individual diseases rather than multimorbidity.^10–13^ A better knowledge of the prevalence and impact of multimorbidity facing COPD patients in primary care would help to evaluate whether a different approach (i.e., multimorbidity) should be taken.

Research on the prevalence of comorbidities among patients with COPD and their impact on health status in primary care patients is scarce. Most studies that evaluated the spectrum and prevalence of comorbidities affecting COPD patients have been conducted in secondary care settings. For example, Divo *et al.*^4^ found a relationship between comorbidities and the risk of death over 51 months. Anxiety, cancers, and heart diseases are among the most significant comorbid diseases associated with COPD mortality risk in secondary care settings.^4^ Increasing knowledge about COPD comorbidities in primary care is essential for the development of better intervention strategies and for reframing clinical guidelines.

This is a unique opportunity to evaluate the impact of individual comorbidities and multimorbidity on COPD patients in primary care in different settings.

## Aims

To determine the prevalence of comorbid conditions in COPD patients, and to assess their association on the health status and on COPD symptoms in primary care patients in a range of settings across Europe. This study will address the following research questions:

1. What is the prevalence of comorbid conditions in COPD patients in real-life primary care?
2. Is there an association of one or more comorbid conditions with health status and with COPD symptoms in different primary care cohorts?

## Methods

### Design

The study involves a cross-sectional analysis of primary care data sets from different countries across Europe. At least eight data sets will be included in the analysis.

### Inclusion/exclusion criteria

Members of the UNLOCK Group of the International Primary Care Respiratory Group (IPCRG)^14^ with cross-sectional data from primary care settings^15,16^ will be invited to participate in the study. Data sets will be eligible for inclusion if they are broadly representative of the primary care settings of the community of origin and include the required variables (see below). Patients should have a general practitioner (GP) diagnosis of COPD.

### Data collection

Required data variables for participation include the following:

- Age
- Gender
- Lung function (i.e., forced expiratory volume in one second (FEV1) (% of predicted) and FEV1/FVC (forced vital capacity)-ratio), if available
- COPD disease-specific health status assessed by the Clinical COPD Questionnaire (CCQ)^17^ and/or by the COPD Assessment Test (CAT)^18^
- Medical Research Council (MRC) dyspnoea scale^19^
- Body mass index
- Smoking status (never, ex-smoker, and current)
- Educational level as a proxy for socio-economic status
- Data on comorbidities
  1. Heart failure, ischaemic heart disease (or ‘heart disease’)
  2. Hypertension
  3. Diabetes
  4. Depression
  5. Asthma
  6. If available: osteoporosis, anxiety, sleep apnoea, rheumatic disease, cancer, rhinitis, stroke, dementia, and anaemia.

Data may be collected in various ways and will be clearly reported and accounted for in the analyses, if appropriate.

### Primary outcome

The primary outcome will be COPD disease-specific health status assessed using the CCQ and/or CAT.

### Data analysis

The cohorts are derived from heterogeneous settings and countries, and therefore the data sets will not be combined, but the results will be analysed and presented separately. Data will initially be presented using descriptive statistics with graphical display. Missing values will be identified and excluded from the analysis with pairwise deletion. Differences between groups (based on the number of comorbid conditions) in health status measures (CCQ and/or CAT) and in COPD symptoms measure (MRC score) will be tested with factorial analysis of covariance (ANCOVA) models adjusting for important potential confounders (e.g., age, sex, smoking status, and disease severity). Estimated marginal means will be compared across data sets.

The *χ*^2^-test will be used to analyse differences in the prevalence of comorbidities. Predictive associations between variables will be studied with binary logistic regression models, and odds ratios and respective confidence intervals (e.g., 95%) will be reported. Statistical significance will be considered for *P* values <0.05. The uncertainty of estimates, whenever possible, will be quantified in terms of confidence interval (e.g., 95%), and effect size values (e.g.,* r*^2^, Partial Eta Squared) will also be analysed, interpreted, and reported. The analysis will be performed by the UNLOCK researcher. Analyses will be performed using SPSS, Version 20.0 (SPSS Inc, Chicago, IL, USA).

A sample size calculation is not applicable here, given that the study will be conducted by comparison of data from different samples that have already been collected. The known sample sizes will provide sufficient statistical power for even the very small effect sizes. For example, the values for a very small effect size such as *d*=0.15 (Cohen’s effect size for means difference) will require a sample of 1,500 patients for 90% statistical power: For *d*=0.35, a sample size of 250 will be required for the same statistical power.

### Ethical approval

All included data sets must be approved by the ethics committees according to national law.

Participants identified in the data sets will remain anonymous, and patient confidentiality will be assured in the collection and merging of the data sets.

## Short discussion

The diagnosis and management of comorbid conditions in COPD has important effects on exacerbations, health status, and mortality.^2–5^ However, there is insufficient knowledge about the prevalence and impact of more than one comorbid condition in COPD patients in primary care settings.^8^ Knowledge of comorbidities in COPD and a better understanding of their impact on health status may help to inform clinical practice and healthcare service delivery.^9,10^

Although recent GOLD updates recognise the need to assess health status and include it together with FEV1 and exacerbations in the management algorithm, suggestions about comorbidities are limited to the recommendation that guidelines should be followed for each disease separately.^11^ However, it is well known that health status is only weakly associated with spirometric values^20^ such as the FEV1, and that some comorbidities as depression and anxiety are the factors that more strongly correlate with health status.^21^ It is expected that this study will show to what extent other diseases or clusters of diseases also influence health status and will consequently inform our approach to COPD management.

In addition, this study of comorbidities will provide new insight into the development of preventive interventions, the reduction of burden of disease, and the adjustment of healthcare services to meet patients’ needs in primary care. Expected results will provide knowledge about the prevalence of comorbidities in COPD, and their impact on health status between genders, age groups, and countries. We also expect that the result from this study, and future research on how and which multiple conditions affect patients’ health status could be used to achieve a truly patient-centred care.
